# Lime Juice Enhances Calcium Bioaccessibility from Yogurt Snacks Formulated with Whey Minerals and Proteins

**DOI:** 10.3390/foods9121873

**Published:** 2020-12-16

**Authors:** Jing Wang, Kataneh Aalaei, Leif H. Skibsted, Lilia M. Ahrné

**Affiliations:** Department of Food Science, University of Copenhagen, 1958 Frederiksberg C, Denmark; jingwang@food.ku.dk (J.W.); kataneh@food.ku.dk (K.A.); ls@food.ku.dk (L.H.S.)

**Keywords:** yogurt snacks, in vitro digestion, citric acid, free calcium, soluble calcium, calcium bioaccessibility

## Abstract

Yogurt-based snacks originally with a calcium content between 0.10 and 0.17 mmol/g dry matter were enriched with a whey mineral concentrate and whey protein isolate or hydrolysate. Whey mineral concentrate was added to increase the total amount of calcium by 0.030 mmol/g dry matter. Calcium bioaccessibility was determined following an in vitro protocol including oral, gastric, and intestinal digestion, with special focus on the effect of lime juice quantifying calcium concentration and activity. Calcium bioaccessibility, defined as soluble calcium divided by total calcium after intestinal digestion amounted to between 17 and 25% for snacks without lime juice. For snacks with lime juice, the bioaccessibility increased to between 24 and 40%, an effect attributed to the presence of citric acid. Citric acid increased the calcium solubility both from whey mineral concentrate and yogurt, and the citrate anion kept supersaturated calcium soluble in the chyme. The binding of calcium in the chyme from snacks with or without lime juice was compared electrochemically, showing that citrate increased the amount of bound calcium but with lower affinity. The results indicated that whey minerals, a waste from cheese production, may be utilized in snacks enhancing calcium bioaccessibility when combined with lime juice.

## 1. Introduction

Snack consumption has increased in recent decades, and this change in dietary patterns has contributed to the current obesity epidemic [1]. Since the majority of the snacks on the market contain excessive amounts of fat, sugar, salt and energy, food research should now focus on developing healthy snacks [2]. One of the important roles of selective snacks is providing balanced energy and essential nutrients such as fiber, proteins, vitamins and minerals in between meals [3]. Yogurts are heathy snacks suitable for in between meals leading to reduced hunger and increased fullness. In comparison with other dairy products, yogurts are more acceptable to lactose intolerant individuals and also have high calcium bioavailability [4].

Calcium is an essential nutrient for humans and dairy products are the primary sources of calcium in the daily diet of many populations. However, low absorption of calcium results in severe calcium malnutrition in many individuals, often leading to osteoporosis [5]. About one third of calcium in milk is water soluble, and the rest is colloidal calcium bound to caseins in micelles [6]. Increasing the amount of soluble calcium is important to promote calcium absorption in the human body. Analytical methods for calcium absorption include in vivo bioavailability and in vitro bioaccessibility digestion models. In vitro methods can provide information on the nutritional impact of food formulations and processing before application of more demanding and expensive in vivo trials [7]. In vitro digestion methods have been widely used compared with in vivo digestion, which have the advantages of lower cost, controllable conditions, better repeatability and convenient sampling [8,9].

Calcium absorption occurs primarily in the intestines. However, the dissociated calcium salts in acidic gastric phase may precipitate in the intestines due to the increasing pH [10]. Complex binding to protein hydrolysates, peptides, and hydroxycarboxylates may enhance calcium absorption and decreases the extent of precipitation, otherwise reducing the free calcium concentration. Therefore, calcium hydroxycarboxylates like calcium citrate are good supplements to ensure optimal absorption for achlorhydria or busy individuals, which can be taken without food [11]. Clinical data have shown that calcium citrate is better in terms of bioavailability than calcium carbonate and tricalcium phosphate, and calcium citrate as supplement seems more safe and with high tolerability to minimize the absorption inhibition of other nutrients [12]. Lime juice is a rich natural source of citric acid, and when combined with calcium hydroxide, the resulting calcium citrate solution has been shown to provide higher ionic calcium concentration and more bioavailable calcium compared to solid calcium citrate [13,14].

Capolac^®^, a by-product of cheese processing, is a natural whey mineral concentrate high in calcium hydrogen phosphate and hydroxyapatite with a composition similar to bone and teeth. Ingredients like Capolac^®^ are increasingly used for calcium fortification of infant’s nutritional products, functional foods and beverages with an optimal calcium-phosphorus ratio for human nutrition [15]. Whey minerals, which have low aqueous solubility, have to become soluble during food digestion, and subsequently available for absorption in the intestines. The addition of hydrogen citrate has been shown to lead to robust supersaturation of whey minerals under the pH condition of the intestines [16].

Whey proteins are considered more effective in promoting the plasma amino acids and protein anabolism especially for the elderly [17,18]. In our previous study, whey protein isolates contributed to the yogurt snacks with the highest calcium bioaccessibility despite a low total calcium content [19]. Furthermore, whey proteins are a source of essential amino acids, and their enzymatic hydrolysates release a wide range of bioactive compounds with health-promoting properties, i.e., antioxidant activity, anti-thrombotic, anti-gastric activities and as a mineral carrier [20,21]. It has also been shown that the chelation of calcium to whey protein hydrolysates exhibits excellent stability and absorbability under both gastric and intestinal conditions using Caco-2 cells, and the main binding sites of calcium was consistent with the positions of carboxyl and carbonyl groups in whey protein hydrolysates [22]. Thereby, whey protein hydrolysates are able to solubilize calcium phosphate and consequently increasing calcium bioaccessibility.

Thus, there is a potential in increasing the bioavailability of calcium in dried foods by combination of whey protein ingredients, minerals and natural acidification of lime juice. However, limited understanding is available regarding the interaction of these components in a food matrix. Therefore, the aim of this study is to understand the improvement of calcium bioaccessibility of freeze-dried yogurt based snacks containing whey minerals, whey protein isolate and whey protein hydrolysate combined with lime juice using an in vitro static digestion method.

## 2. Materials and Methods

### 2.1. Materials

Natural yogurt (Arla Foods, Viby J, Denmark) with 0.5% fat and 5.5% protein according to the manufacturer, organic lime juice (100% fruit content, Voelkel GmbH, Höhbeck, Germany) and sucrose (Nordic Sugar A/S, København K, Denmark) were purchased locally.

Whey protein isolate (Lacprodan^®^ DI-9224, containing 6.0% moisture, 4.5% ash, 0.2% lactose and 0.2% fat), Peptigen^®^ IF-3080 as whey protein hydrolysates (Degree of hydrolysis 23–29%) and Capolac^®^ MM-0525 BG were the products of Arla Foods Ingredients Group P/S (Viby J, Denmark). Capolac^®^ is a natural whey mineral concentrate, which contains 24.0% calcium, 12.5% phosphorus, 8.0% lactose, 6.0% moisture, less than 3.0% protein and less than 1.0% fat, according to the product specification. Pectin (GENU^®^ Pectin type YM-115-L, DE~72%) was obtained from CP Kelco ApS (Lille Skensved, Denmark). Milli-Q water (Millipore Corporation, Bedford, MA, USA) was used to prepare all solutions.

### 2.2. Liquid Chromatographic Analysis of Citric Acid in Lime Juice

Lime juice samples were centrifuged at 5000× *g* for 10 min at 4 °C, filtered using syringe filters (0.2 µm, VWR International, PR, USA) and diluted ten times with Milli-Q water for analysis. A HPLC method was developed using a 1200 series HPLC system equipped with a quaternary pump and a thermostatted autosampler (Agilent Technologies, Santa Clara, CA, USA). Citric acid was quantified in triplicate using a diode array detector (Agilent 1100/1200) monitored at 210 nm and an Aminex HPX-87H ion exclusion column at a controlled temperature of 55 °C. The analytical conditions were as follows: mobile phase 0.045 N H_2_SO_4_/acetonitrile (94:6; *v*/*v*), flow 0.3 mL/min, and detector temperature 35 °C [23].

### 2.3. Yogurt Base Preparation

Yogurt, pectin and sucrose combined with whey protein isolate (WPI), whey protein hydrolysate (WPH), whey mineral concentrate (WMC) or lime juice were used as yogurt snack bases. Stock solutions of pectin, WPI, WPH, and WMC, were prepared with Milli-Q water to concentrations of 2.0% (*w*/*w*), 8.0% (*w*/*w*), 8.0% (*w*/*w*) and 1.4% (*w*/*w*), respectively. The effective pectin content of the above pectin solution was 1.0% as the pectin preparation containing about 50% sucrose. The yogurt bases were prepared in duplicate and then homogenized (T25 Basic, IKA-Werke GmbH & Co, Staufen, Germany) at 9500 r/min for 1 min to get homogenous mixtures, and labelled as Y, Y/I, Y/H, Y/C, Y/I/C, and Y/H/C (L indicated lime juice addition) as shown in Table 1 according to different formulations. The pH of yogurt bases was characterized in triplicate at room temperature by HQ411d pH meter from Hach Company (Loveland, CO, USA).

### 2.4. Freeze Drying and Snack Composition

The round silicone molds (Micro Round 5 Silikoneform, Venezia, Italy) of 2.4 cm diameter and 1.2 cm height were used for shaping snacks. After pre-freezing overnight at −80 °C, the samples were dried by a vacuum freeze drier (Modulyo, Edwards High Vacuum International, Crawley, UK) at −50 °C for 24 h. The cylindrical snacks were weighed to calculate water removal and stored in sealed bags under temperature control (20 °C ± 1 °C) for further analysis. After freeze drying, the snacks appeared crispy and were not hygroscopic.

Protein content (N × 6.38) [24] of yogurt snacks was measured by the elemental analyzer (vario MACRO cube, Elementar Analysensysteme GmbH, Hanau, Germany). Water activity (AquaLab CX-2, Decagon Devices, Inc., Pullman, WA, USA) of snacks was quantified at room temperature after 24 h of equilibrium. Dry matter content (DM) of snacks was quantified by drying snacks in round aluminum containers (Ø 94.66 mL, Plus Pack, Odense, Denmark) in an oven (FED 53, BINDER GmbH, Tuttlingen, Germany) at 103 °C until constant weight [25]. All the analyses were conducted in triplicate.

### 2.5. In Vitro Static Digestion

The static in vitro digestion was performed in duplicate based on the COST INFOGEST standardized method, which simulated oral, gastric and intestinal phase. The simulated salivary fluid (SSF), simulated gastric fluid (SGF) and simulated intestinal fluid (SIF) were prepared according to the COST INFOGEST protocol and stored at −20 °C, and preheated to 37 °C before use [9].

Briefly, snacks were first grinded (Coffee Mill 2393, OBH Nordica, Copenhagen, Denmark), and then 2.0 g snacks were mixed with 18 mL of SSF and incubated in a 37 °C water bath (Julabo EH-19, Seelbach, Germany) with stirring speed of 200 rpm for 2 min to simulate oral phase.

A total of 15 mL of SGF, 10 µL of 0.3 mol/L CaCl_2_ and 0.59 mL Milli-Q water were added in turn. The pH was adjusted to 3.0 with 1.0 mol/L HCl to simulate gastric environment. Porcine pepsin (P7012, Sigma-Aldrich Co., St. Louis, MO, USA) was completely dissolved in SGF, and 4.0 mL of pepsin stock solution were added to the final 40 mL mixture to achieve 2000 U/mL. The simulated gastric phase was stirred for 2 h at 37 °C with a stirring speed of 200 rpm. The pH and free calcium concentration (see below) were checked every 30 min.

Then, 15 mL of gastric mixture was mixed with 6.39 mL SIF, 30 µL of 0.30 mol/L CaCl_2_, 1.044 mL of Milli-Q water and 1.89 mL of bile to simulate intestinal phase. Bile (B8631, Sigma-Aldrich Co., St. Louis, MO, USA) was dissolved with Milli-Q water to reach 10 mmol/L in the final 30 mL mixture. The pH was adjusted to 7.0 with 1.0 mol/L NaOH. Pancreatin (P7545, Sigma-Aldrich Co., St. Louis, MO, USA) was dissolved in SIF, and 5.0 mL of pancreatin stock solution was added to the final intestinal mixture in order to reach trypsin activity of 100 U/mL. The simulated intestinal mixture was stirred at 37 °C for 2 h. The free calcium concentration (see below) was tested every 30 min.

10 mL intestinal mixture were sampled at the beginning (*t* = 120) and at the end (*t* = 240) of the intestinal phase during digestion. A total of 500 µL of Pefabloc (1 mmol/L, 76307, Sigma-Aldrich Co., St. Louis, MO, USA) were added to inhibit trypsin activity. All samples were frozen at −80 °C for further chemical analysis.

### 2.6. Calcium Analysis

#### 2.6.1. Free Calcium

An ion selective electrode ISE25Ca (Mettler Toledo, Schwerzenbach, Switzerland) with a reference electrode REF251 (Hach Company, Loveland, CO, USA) was used to determine calcium ion activity. Different standard concentration of CaCl_2_ (1.0 × 10^−4^, 1.0 × 10^−3^ and 1.0 × 10^−2^ mol/L) was used to calibrate the electrode at 37 °C as the temperature of digestion. The free calcium concentration was converted to the amount of free calcium available per gram dry matter of snack. The standard curve was obtained according to the linear regression between the electric potential (mV) and − log (*α*Ca^2+^) [26]:

(1) $$\alpha {\mathrm{Ca}}^{2+}=c_{{\mathrm{CaCl}}_{2}}\times \;\gamma {\mathrm{Ca}}^{2+}$$

where *α*Ca^2+^ is the calcium ion activity and *γ*Ca^2+^ is the calcium activity coefficient based on the Davies equation:

(2) $$\log\gamma {\mathrm{Ca}}^{2+}=\;-\;A_{DH}z^{2}\left(\frac{\sqrt{I}}{1\;+\;\sqrt{I}}\;-\;0.30I\right)\;$$

where *A_DH_* is the Debye–Hükel constant of 0.519 at 37 °C, *z* is charge of calcium ion and *I* is the ionic strength.

For the samples with unknown ionic strength, electrical conductivity was measured using a multimeter sensION + EC71 (Hach lange S.L.U., Barcelona, Spain) with a Sension + 5070 conductivity cell (Mettler Toledo, Schwerzenbach, Switzerland). Ionic strength was calculated according to Ponnamperuma, et al. [27]:

(3) I=16EC . 

where *EC* is the electrical conductivity in mS/cm.

#### 2.6.2. Total Calcium

Snack samples, WPI, WPH and WMC were all microwaved (UltraWAVE, Milestone Srl, Sorisole, Italy) prior to analysis to ensure that all organic matter was digested. Briefly, 100 mg samples were digested at 240 °C with 2.5 mL of 70% HNO_3_ and 1.0 mL of 15% H_2_O_2_ with Milli-Q water to 50 mL. Total calcium content was analyzed in triplicate by ion coupled plasma optical emission spectroscopy (5100 ICP-OES, Agilent Technologies, Santa Clara, CA, USA) at the wavelengths of 318.1 nm.

#### 2.6.3. Soluble Calcium

The samples of in vitro intestinal digestion were centrifuged at 8000× *g* for 20 min at 4 °C and pre-filtered using syringe filters (0.2 µm cellulose acetate membrane, VWR International, Puerto Rico, PR, USA). Ultrafiltrates were obtained by centrifugal ultrafiltration (molecular weight cut off 3 kDa, VIVASPIN 20, Sartorius Stedim Lab Ltd., Stonehouse, UK) at 6000× *g* for 40 min [28]. A total of 1.0 mL of ultrafiltrated sample was diluted to 10 mL with 5% HNO_3_ followed by ICP-OES analyses to get soluble calcium content. In vitro calcium bioaccessibility was calculated according to:

(4) $$\mathrm{calcium}\mathrm{bioaccessibility}\left(\%\right)=\frac{\mathrm{soluble}\mathrm{calcium}\left(\mathrm{mmol}/g\mathrm{DM}\right)}{\mathrm{total}\mathrm{calcium}\mathrm{in}\mathrm{snacks}\left(\mathrm{mmol}/g\mathrm{DM}\right)}\times 100$$

where soluble calcium was converted to the amount of soluble calcium available per gram dry matter of snack.

### 2.7. Calcium Binding Measurements

Determination of calcium binding was based on the total calcium concentration of snacks as quantified by ICP-OES and free calcium concentration of digestive juice measured by the calcium electrode. Briefly, the selective snacks Y and Y/L were digested by static in vitro digestion protocol as mentioned above. After 2 h gastric digestion, the pH was adjusted to 7.0. Then the free calcium content in 40 mL samples of the digestive juice was measured at 37 °C after each addition of 100 μL of 0.50 mol/L CaCl_2_, and the total calcium concentration was corrected after calcium chloride addition. The total calcium minus the free calcium gave a value for the bound calcium content. Two independent experiments were applied. The protein content was expressed as gram due to the complexity of the mixture of digestive juice. The Klotz method was used to estimate the binding constant of calcium to the biopolymers (proteins, polysaccharides and hydroxycarboxylates) present in the digested snacks, and the binding constant was calculated according to the following equation:

(5) $$1/B=\left(1/nK\right)\left(1/\left[{\mathrm{Ca}}^{2+}\right]\right)+1/n\;$$

where *B* is the bound calcium per gram of protein mixture, [Ca^2+^] is the free calcium concentration determined electrochemically, *K* is the binding constant, and *n* is the number of moles of calcium bound per gram of protein mixture [29].

### 2.8. Dissolution of WMC by Lime Juice

A series of dissolution experiments with whey mineral concentrate, whey protein isolate, whey protein hydrolysate or lime juice were performed in duplicate in order to determine the importance of citric acid to dissolve whey mineral concentrate. The concentration was the same as the yogurt base of snacks: WMC 0.098% (*w*/*w*), WPI 2.8% (*w*/*w*), WPH 2.8% (*w*/*w*) and lime juice 3% (*w*/*w*). WMC was firstly equilibrated for 2 h at room temperature. Then, the ingredients were added as set: WMC, WMC/WPI, WMC/WPH with or without lime juice. The pH was adjusted to 3.0 with 6.0 mol/L HCl and 7.0 with 2.0 mol/L NaOH, respectively, to simulate gastrointestinal environment. Samples were incubated with magnetic stirring at 600 rpm, and turbidity and free calcium concentration (see above) were measured over 48 h. Turbidity was measured trough a 25 mm cell at room temperature with an infrared light turbidity meter (AL450T-IR, Aqualytic, Dortmund, Germany) [16].

### 2.9. Statistical Analysis

The statistical differences among the compositions and lime juice addition were subjected to the one-way ANOVA analysis (SPSS 25, SPSS Inc., Chicago, IL, USA). The significant level *p* < 0.05 was used with the Tukey’s test.

## 3. Results and Discussion

### 3.1. Characterization of Snacks

Different formulations of yogurt bases were prepared as shown in Table 1, all made by natural ingredients from milk production combined with lime juice to form crispy calcium rich snacks. Natural yogurt was used as dairy base since it is richer in milk proteins and calcium than most other liquid milk products, and the acid pH is favorable for calcium solubility. The snacks were enriched with WPI and WPH because they have better nutritive value as an important source of protein fragments and bioactive peptides. Citrus fruits such as lime and their juice have a high content of vitamin C, carotenoids, phenolic compounds and citric acid, which all provide health benefits, and lime juice was selected for the actual formulations as the most acidic of the citrus fruits [30]. The citric acid concentration in the lime juice used was 50 mg/mL, and the juice was added to yogurt bases to yield 1.50 mg/mL. As can be seen in Table 1, the pH of yogurt bases with lime juice were significantly lower than of the other samples due to the high acid content of lime juice and the low pH of 2.28. In other words, lime juice decreased the pH from 4.29 (4.41) to 4.01 (4.13) for the samples with 70% yogurt content like Y and Y/C. However, the pH decreased even more for the other samples with 35% yogurt after adding lime, as a consequence of the buffer capacity of the ingredients in yogurts such as the caseins. The pH of yogurt, WPI solution (8.0%), WPH solution (8.0%) and pectin solution (8.0%) was 4.38, 6.80, 6.92 and 3.79, respectively, which led to a lower pH for the samples containing 70% yogurt, such as Y and Y/C, compared to the other samples.

Freeze-drying resulted in removal of 80–83% water from the yogurt base, which was not influenced by the composition of the yogurt bases. The dry matter content of the yogurt snacks amounted to 98%. Water activity (a_w_) controls microbial growth and food stability, and there is normally no microbial proliferation when a_w_ is less than 0.61 [31]. Water activity was found to be between 0.305 and 0.357, which indicates that snacks can be kept at room temperature without microbial growth. Therefore, the yogurt snacks in the current study are expected to have good shelf life due to the low moisture content and water activity. As expected, protein content of the snacks was positively correlated to the concentration of yogurt, WPI and WPH addition. Y/I has the protein content of 24% that is higher than Y/H (22%) and Y (20%), because of the higher protein content in WPI (85%) compared to WPH (79%). Added lime juice and WMC did not influence the protein content of the snacks.

### 3.2. Calcium Analysis

#### 3.2.1. Total Calcium Concentration of Yogurt Snacks

**Figure 1:**
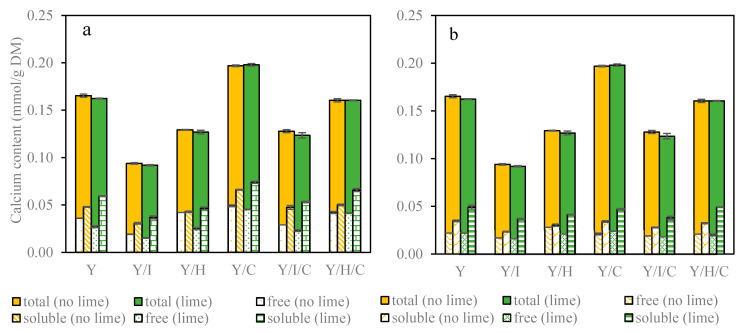
Calcium content per gram dry matter of snacks by adding lime juice (**a**) at the beginning; (**b**) at the end of intestinal phase.

Total calcium concentration is the total amount of all forms of calcium in the samples, which varied according to the formulations as shown in Figure 1. The total calcium content of WMC, natural yogurt, WPH and WPI was 6.69 mmol/g DM, 0.39 mmol/g DM, 0.22 mmol/g DM and 0.012 mmol/g DM, respectively. As expected, whey mineral concentrate served as a natural calcium supplement and increased the total calcium content of the snacks significantly. Notably, the whey protein hydrolysates had 18 times more calcium content than whey protein isolates, a difference also affecting the final calcium content of the snacks.

The calcium content in the snacks was found to depend on the composition of yogurt bases, and was 0.165 ± 0.002 mmol/g DM, 0.094 ± 0.001 mmol/g DM and 0.129 ± 0.002 mmol/g DM in Y, Y/I and Y/H, respectively. Y/I and Y/I/L had the lowest amount of total calcium in comparison to the other snacks, because WPI contained only a little calcium. WMC addition increased calcium content, as expected, by 0.030 mmol/g DM in all snacks, and resulted in the highest total calcium concentration of 0.197 ± 0.001 mmol/g DM in Y/C. The snacks Y/I/C and Y/H/C had calcium content of 0.128 ± 0.003 mmol/g DM and 0.160 ± 0.002 mmol/g DM, respectively. Lime juice addition, as well as pectin and sucrose, had no effect on total calcium content.

#### 3.2.2. Free Calcium Concentration during In Vitro Digestion

**Figure 2:**
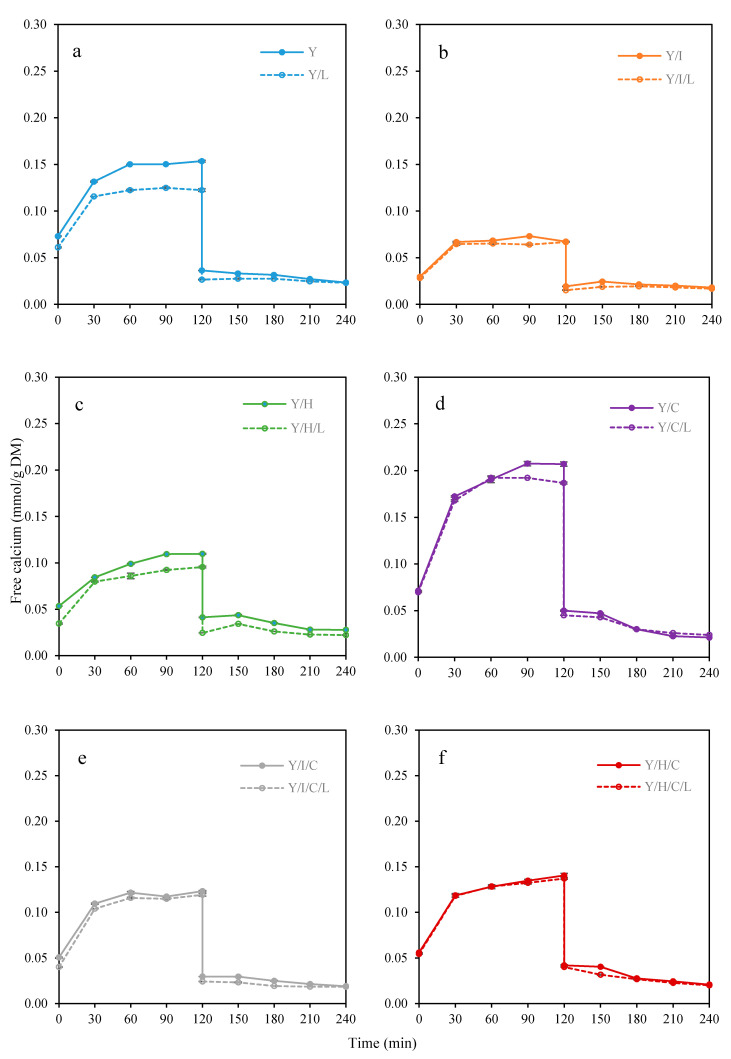
Free calcium changes per gram dry matter of snacks during in vitro digestion, including gastric phase at pH 3 (0–120 min) and intestinal phase at pH 7 (120–240 min). (**a**) Y and Y/L; (**b**) Y/I and Y/I/L; (**c**) Y/H and Y/H/L; (**d**) Y/C and Y/C/L; (**e**) Y/I/C and Y/I/C/L; (**f**) Y/H/C and Y/H/C/L.

In vitro models are often the efficient tools for pre-evaluation of numerous variables prior to clinical trials [8], and therefore used in this study to compare the different snack formulations. Free calcium concentration as ionized calcium during gastrointestinal digestion, was quantified by calcium selective electrodes and expressed as mmol/g DM of snacks to be comparable with total calcium content. Changes in free calcium concentration of the yogurt snacks for 240 min of oral, gastric and intestinal in vitro digestion are presented in Figure 2.

In the short oral period, yogurt snacks were partially dissolved and then transferred to gastric digestion. At the beginning of the gastric phase, the free calcium content of Y (0.071 ± 0.002 mmol/g DM) was significantly higher than Y/I (0.029 ± 0.000 mmol/g DM) and Y/H (0.053 ± 0.001 mmol/g DM). The addition of lime juice reduced the initial free calcium in Y/L (0.060 ± 0.001 mmol/g DM) and Y/H/L (0.034 ± 0.001 mmol/g DM), but had no significant effect on Y/I/L (0.028 ± 0.001 mmol/g DM). The addition of WMC significantly increased the initial free calcium content of snacks containing lime juice by between 15 and 59%. During the gastric digestion, the free calcium content almost doubled in the first 30 min, and then changed slightly during 30 min to 120 min of gastric digestion. At the end of gastric phase, the free calcium of Y (0.150 ± 0.001 mmol/g DM) was more than for Y/I (0.066 ± 0.000 mmol/g DM) and Y/H (0.109 ± 0.001 mmol/g DM). For snacks containing lime juice, the final free calcium in Y/L and Y/H/L decreased by 20 and 15%, respectively, but was changed only a little in Y/I/L (0.065 ± 0.002 mmol/g DM). The snacks with the added WMC had higher final free calcium like Y/C (0.197 ± 0.002 mmol/g DM), Y/I/C (0.122 ± 0.001 mmol/g DM) and Y/H/C (0.141 ± 0.002 mmol/g DM) than those without WMC, which indicated WMC released free calcium during gastric phase under acidic conditions. The free calcium concentration of the snacks with lime juice was lower than the snack without lime juice in all cases during gastric digestion. This could be explained by binding of ionic calcium by hydroxycarboxylates, like citrate even at low pH, which consequently caused a reduction of free calcium content.

Paracellular calcium absorption depends on the concentration of vitamin D and free calcium in the intestines [32]. Therefore, the amount of free calcium in intestinal digestion is important since free calcium is in equilibrium with soluble calcium. At the early intestinal phase (120 min), the free calcium concentration was substantially reduced by 85–89% in all samples at pH 7.0. pH is the most critical factor affecting ionic calcium content, and Goss et al. [33] confirmed that calcium salts such as calcium citrate tetrahydrate, calcium phosphate and calcium glycerophosphate showed a decrease of solubility as the pH increased from the acidic environment of the stomach to the neutral pH of the intestine. Competition between calcium precipitation and complex binding determines the concentration of free calcium ions in the intestine [34]. At the beginning of intestinal phase, Y/H (0.042 ± 0.002 mmol/g DM) had higher free calcium than Y (0.036 ± 0.000 mmol/g DM) and Y/I (0.019 ± 0.000 mmol/g DM), and snacks containing lime juice had lower free calcium concentration between 21 and 40%. WMC addition increased free calcium by 0.010 mmol/g DM except for the snack with added WPH, and Y/H/C had the same free calcium concentration as Y/H in the initial phase of intestines. As digestion in the intestinal phase proceed, the free calcium concentration continued to decrease. At the end of intestinal digestion, the free calcium content in Y/H (0.028 ± 0.000 mmol/g DM) was higher than in Y (0.022 ± 0.000 mmol/g DM) and in Y/I (0.017 ± 0.000 mmol/g DM). The addition of WMC was seen to have little effect on the final free calcium concentration. After 240 min of intestinal digestion, free calcium content of samples with WMC was decreased by 50% compared to the beginning of the intestinal phase because of precipitation of calcium phosphate, i.e., Y/C (0.021 ± 0.001 mmol/g DM), Y/I/C (0.019 ± 0.000 mmol/g DM) and Y/H/C (0.021 ± 0.000 mmol/g DM), respectively. Addition of lime juice slowed the loss of free calcium during the intestinal digestion for all samples. At the end of digestion, Y/C was the only sample having higher free calcium with lime juice added (0.024 ± 0.000 mmol/g DM) than that without lime juice added (0.021 ± 0.001 mmol/g DM), which could be explained by continuing supersaturation of calcium dissolved from WMC by citric acid.

Overall, free calcium concentration was positively correlated with total calcium concentration in static in vitro digestion experiments. Y/H had higher free calcium than Y/I due to the higher calcium content of WPH in comparison to WPI. In the gastric phase, free calcium increased from the beginning to the end of digestion by a factor of two, which was independent of the presence of lime. Lime juice reduced the concentration of free calcium both in the initial and final digestion. Calcium binding to organic molecules like hydroxycarboxylates prevents calcium precipitation, but it also reduces spontaneous diffusion caused the decrease of free calcium content [35]. During intestinal digestion, free calcium decreased in all cases, but most in the presence of WMC and less significantly in the presence of WPI. There was no significant difference of free calcium concentration with lime addition at the end of intestinal phase, except by the presence of WPH for which the free calcium decreased by 25%, while for the snack with WMC added, the free calcium increased by 14%.

#### 3.2.3. Soluble Calcium Concentration and Calcium Bioaccessibility

Concentration of soluble calcium was measured in ultrafiltrates from intestinal digestion samples, which is a protein-free phase [36]. Soluble calcium content per gram dry matter of snacks is presented in Figure 1. At the beginning of intestinal phase (Figure 1a), there was a higher amount of soluble calcium in Y (0.048 ± 0.000 mmol/g DM) than Y/I (0.031 ± 0.001 mmol/g DM) and Y/H (0.043 ± 0.001 mmol/g DM). Lime juice addition increased soluble calcium concentration significantly by approximately 20% of Y/L and Y/I/L, but only by 7% of Y/H/L. When WMC was added to snacks, soluble calcium was increased by a factor of 1.4 except for Y/H/C, which had an increase by a factor of 1.2 as the largest difference between Y/H/C and Y/H/C/L.

As the digestion proceeds to the end of intestinal phase as shown in Figure 1b, soluble calcium decreased (except Y/I/L) compared to the initial intestinal phase, and lime juice addition made this reduction smaller. The effect of citric acid on the solubility of calcium salts in dairy products appears to be important for the transformation of calcium forms during digestion. This transformation between amorphous calcium phosphate and precipitated calcium citrate is related to the feature of calcium citrate as an improver of calcium absorption, since the long lag phase of calcium citrate precipitation leads to adequate absorption of calcium in the intestine [35]. Y (0.035 ± 0.001 mmol/g DM) also had higher final soluble calcium than Y/I (0.023 ± 0.000 mmol/g DM) and Y/H (0.030± 0.001 mmol/g DM). However, WMC addition had little effect on final soluble calcium, except for the samples Y/I/C and Y/H/C/L, in which soluble calcium increased by around 20%. Lime juice enhanced final soluble calcium between 36 and 61% probably due to supersaturation, with Y/I/L showing a larger difference than Y/I.

The difference between soluble calcium and free calcium represents complexed bound calcium [36]. The complex bound calcium as presented in Figure 1 was around 0.010 mmol/g DM for Y and Y/I, but only 0.0010 mmol/g DM for Y/H in the intestinal phase. Whey protein hydrolysates may have few ligands suitable for calcium binding during digestion. The presence of WMC increased complex bound calcium for Y/C and Y/I/C to 0.020 mmol/g DM, and for Y/H/C to 0.010 mmol/g DM. Furthermore, citric acid addition resulted in the complex bound calcium two to three times higher than for the snacks without citric acid added during intestinal digestion, and consequently caused an increase of soluble calcium and accordingly of calcium bioaccessibility. The presence of suitable ligands like hydroxycarboxylates is crucial for calcium complexation when the digestive food is moved from the stomach to the intestines. Complex bound calcium improves calcium accessibility by preventing precipitation of calcium salts in neutral conditions of the intestines [34].

**Figure 3:**
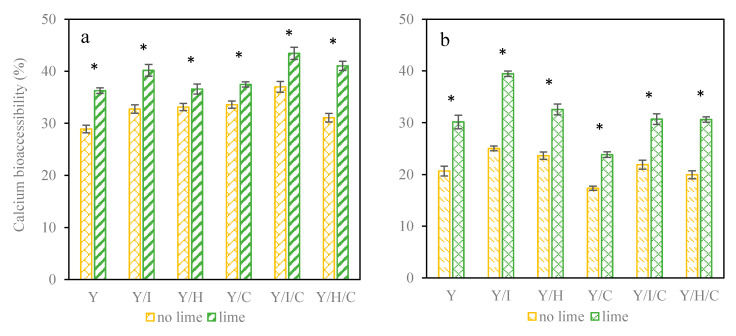
Calcium bioaccessibility (**a**) at the beginning; (**b**) at the end of intestinal phase. * indicated significant differences of lime juice addition in Tukey (*p* < 0.05).

Bioaccessible calcium is the fraction of calcium in the soluble form that is available for its subsequent absorption in the intestine, which is related to total calcium concentration [37]. In the current study the calcium bioaccessibility was 29–43% at the beginning of intestinal phase (Figure 3a), and it was significantly improved when citric acid was added. At the end of intestinal digestion in Figure 3b, calcium bioaccessibility of snacks without citric acid was 17–25%, and bioaccessibility for snacks with citric acid was 24–40%. Recombination of calcium and hydroxycarboxylate complexes in the intestine seems accordingly faster than precipitation confirming the high bioavailability of calcium hydroxycarboxylates [32]. The presence of WPI contributed to the highest final calcium bioaccessibility (Y/I 25% and Y/I/L 40%), despite the lowest soluble and total calcium content. The calcium bioaccessibility decreased from the beginning to the end of intestinal phase except for Y/I/L, which remained at 40%. In addition, the presence of citric acid reduced the decrease in bioaccessibility by 40%.

**Figure 4:**
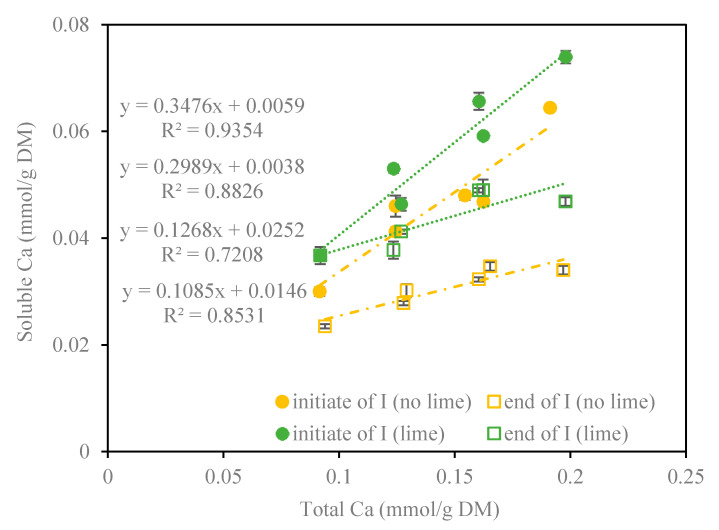
Relationship of soluble calcium versus total calcium per gram dry matter of snacks with or without lime juice addition at the beginning and at the end of intestinal digestion.

Figure 4 shows the relationship between soluble calcium and total calcium in the intestinal digestion phase as a result of lime juice addition. Linearity between soluble calcium and total calcium is confirmed, and the slope of this correlation could reflect the level of calcium bioaccessibility, at least partly. The slope of the correlation for the snacks without lime changed from 0.30 at the beginning to 0.11 at the end of intestinal period while the slope for the snacks with lime decreased from initial 0.35 to final 0.13 during intestinal digestion. This reduction of soluble calcium depends on precipitation of soluble calcium after it is released from the food matrix under in vitro digestion. However, this part of soluble calcium would be available for intestinal absorption but with competition by precipitation under in vivo digestion [19]. The presence of lime juice increased the slope by 16% both for the initial and final intestine phase, an increase that indicates that calcium hydroxycarboxylate formation is faster than calcium precipitation, leading to a higher bioaccessibility.

### 3.3. Calcium Binding Measurements at the Beginning of Intestinal Digestion

**Figure 5:**
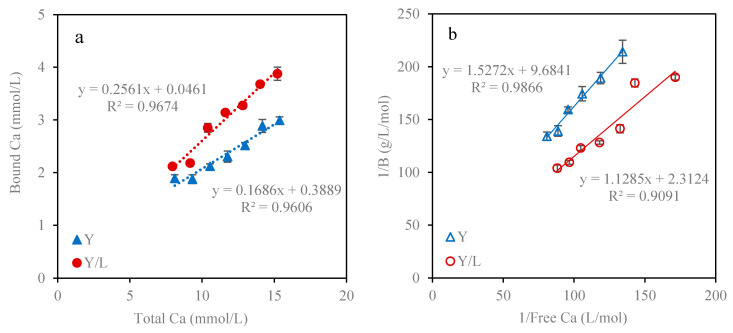
Calcium binding measurements in Y and Y/L snack at pH 7.0 and 37 °C (**a**) The relationship between bound Ca and total Ca; (**b**) Klotz plot analysis for the binding constant calculation. B represents the bound Ca to the per gram of protein.

The binding calcium to proteins and other macromolecules like polysaccharides and hydroxycarboxylates has large influence on calcium absorption during the food digestion process. Tang and Skibsted [29] found the calcium binding affinity was affected by pH and amino acids, and found aspartate as the strongest calcium binder among the studied amino acids at pH 7.4 and 25 °C. Dairy proteins are enzymatically digested and release small molecules with free binding sites for metal ions during in vitro digestion. Furthermore, pectin and citric acid in yogurt snacks also provided calcium binding sites. Calcium binding to Y and Y/L formulations were measured at the beginning of intestinal digestion at pH 7.0 and 37 °C by adding CaCl_2_. It was observed that the total calcium, free calcium and bound calcium content in digestive juice were all increased after the addition of CaCl_2_. The binding capacity of Y and Y/L was 17 and 26% as shown in Figure 5a, respectively, and the addition of lime juice increased the binding capacity by 52% due to the binding of Ca to citrate. Klotz analysis was applied to study the calcium binding constant for calcium and polymers combination as seen in Figure 5b. The number of moles of calcium bound on per gram of protein (*n*) of Y and Y/L was 0.10 and 0.43 as calculated by Equation (5). Surprisingly, Y had a higher calcium binding affinity with the binding constant of 6.34 L/mol compared with Y/L with the binding constant of 2.05 L/mol. The results indicate that citric acid addition results in increasing calcium binding in the intestines, but also that the binding is weaker. Calcium binding to proteins and other ligands like carboxylates at neutral pH could prevent later reprecipitation of calcium released in the stomach, and consequently improve the overall calcium bioavailability [34].

### 3.4. Dissolution of WMC by Lime Juice in Presence of WPI or WPH

**Figure 6:**
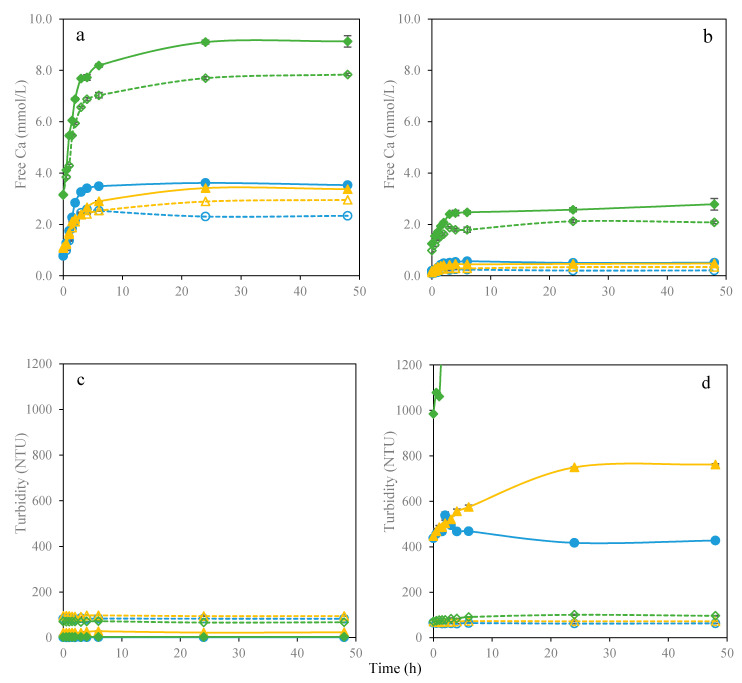
Dissolution of whey mineral concentrate by lime juice for 48 h. (**a**) Free calcium changes at pH 3; (**b**) Free calcium changes at pH 7; (**c**) Turbidity changes at pH 3; (**d**) Turbidity changes at pH 7. Different formulations were expressed as follows: WMC (●), WMC/lime (○), WMC/WPI (▲), WMC/WPI/lime (△), WMC/WPH (◆) and WMC/WPH/lime (◇).

To further understand the effect of citric acid on the dissolution of whey mineral concentrate, simple solutions consisting the mixtures of WMC, WPI and WPH combined with lime juice were prepared. The total calcium content in solutions was 6.56 mmol/L (WMC and WMC/lime), 6.84 mmol/L (WMC/WPI and WMC/WPI/lime) and 12.72 mmol/L (WMC/WPH and WMC/WPH/lime), respectively. At pH 3.0, which is typical pH of the stomach, free calcium concentration increased rapidly during the two hours of stirring as shown in Figure 6a. The free calcium of WMC/WPH (9.1 mmol/L) and WMC/WPH/lime (7.8 mmol/L) was significantly higher than of the other solutions. Whey protein hydrolysates that may be able to solubilize calcium phosphate and consequently increase the content of dissolved calcium. Calcium was solubilized at low pH of the stomach, but precipitated at the higher pH of intestine. At pH 7.0, the free calcium content was reduced by a factor of 8 for WMC, WMC/lime, WMC/WPI and WMC/WPI/lime, but it was only decreased by a factor of 3.5 for WMC/WPH and WMC/WPH/lime (Figure 6b). Whey protein hydrolysates may accordingly prevent ionic calcium precipitating under the neutral condition of the intestines. The presence of citric acid lower the free calcium concentration both under acid and neutral conditions, as seen from the in vitro digestion results.

The increase of protein aggregation and presence of undissolved salts increase the turbidity [38]. From the experiments monitoring turbidity of solutions, turbidity was quite low at pH 3.0 due to the dissolution of calcium phosphate in Figure 6c. Lime juice addition increased the turbidity. When the pH was adjusted to 7.0, the turbidity of solutions without lime, i.e., WMC, WMC/WPI and WMC/WPH increased by a factor of 400 as seen in Figure 6d. However, pH had little effect on turbidity of solutions with lime juice even after 48 h. Garcia, Vavrusova and Skibsted [35] also found the precipitation was not immediate when conditions favored the formation of calcium citrate. WMC as calcium phosphate has low solubility under neutral condition, but can be solubilized by addition of citric acid and citrate from lime juice leading to supersaturation of calcium citrate. This dissolution experiment confirmed that calcium bioaccessibility could be improved also for WMC due to overshooting of calcium hydroxycarboxylate dissolution. Calcium citrate will show a greater absorption, which is attributed to a superior solubility compared to calcium phosphate and calcium carbonate resulting from the persistent supersaturation [15,33].

## 4. Conclusions

Whey mineral concentrate, a side stream from cheese production, increased soluble calcium available from yogurt-based snacks during in vitro static digestion. Added lime juice as a source of citric acid slowed down the loss of free calcium due to precipitation during intestinal digestion otherwise competing with calcium absorption increasing calcium bioaccessibility. Citric acid enhanced calcium solubility both from whey mineral concentrate and yogurt, and the citrate anion combined with free calcium kept supersaturated calcium soluble in the chyme. After adding citric acid, binding of calcium to the chyme increased but with less affinity. Citric acid improved calcium dissolution from whey minerals at the lower pH, and, more importantly, protected calcium supersaturation by citrate following pH increase in the intestines. Yogurt snacks, including the combination of whey mineral concentrate and lime juice, could be marketed by dairy industry as healthy and based on circular economy with a green image.

## Figures and Tables

**Table 1:** Formulation and pH of yogurt bases.

Sample	Yogurt	Sucrose	Pectin Solution (2.0%)	WPI Solution (8.0%)	WPH Solution (8.0%)	WMC Solution (1.4%)	H_2_O	Lime Juice	pH
	%	%	%	%	%	%	%	%	/
Y	70	10	10	/	/	/	10	/	4.29 ± 0.01 *
Y/L	70	10	10	/	/	/	7	3	4.01 ± 0.00 *
Y/I	35	10	10	35	/	/	10	/	4.83 ± 0.03 *
Y/I/L	35	10	10	35	/	/	7	3	4.29 ± 0.01 *
Y/H	35	10	10	/	35	/	10	/	4.93 ± 0.02 *
Y/H/L	35	10	10	/	35	/	7	3	4.44 ± 0.02 *
Y/C	70	10	10	/	/	7	3	/	4.41 ± 0.01 *
Y/C/L	70	10	10	/	/	7	/	3	4.13 ± 0.00 *
Y/I/C	35	10	10	35	/	7	3	/	4.98 ± 0.02 *
Y/I/C/L	35	10	10	35	/	7	/	3	4.43 ± 0.01 *
Y/H/C	35	10	10	/	35	7	3	/	5.09 ± 0.03 *
Y/H/C/L	35	10	10	/	35	7	/	3	4.57 ± 0.01 *

* indicated significant differences of lime juice addition in Tukey (*p* < 0.05).
